# Identifying subgroups of nurses’ voice behavior in clinical settings: a latent profile analysis of work motivation and demographic predictors

**DOI:** 10.3389/fpsyg.2026.1732216

**Published:** 2026-02-26

**Authors:** Jingting He, Yanping Ying, Qiufang Lu, Huihan Zhao, Yuan Wen, Hongyu Lu, Jiajie Ning, Pengxin Dong, Liying Liu

**Affiliations:** 1 Nursing Quality Control Center, The First Affiliated Hospital of Guangxi Medical University, Nanning, China; 2 The Second Ward of Hematology Department, The First Affiliated Hospital of Guangxi Medical University, Nanning, China; 3 Department of Emergency, Jiangbin Hospital of Guangxi Zhuang Autonomous Region, Nanning, China; 4 Department of General Surgery, Guangxi Zhuang Autonomous Region Maternity and Child Health Care Hospital, Nanning, China; 5 School of Nursing, Guangxi Medical University, Nanning, China; 6 The Nursing Department, The Second Affiliated Hospital of Guangxi Medical University, Nanning, China

**Keywords:** latent profile analysis, nurses’ voice behavior, nursing management, self-determination theory, work motivation

## Abstract

**Introduction:**

Nurses’ voice behavior is critical for patient safety and organizational improvement. However, its manifestation is not uniform among nurses. This study aimed to identify latent profiles of nurses’ voice behavior using Latent Profile Analysis (LPA) to understand this heterogeneity and explore its influencing factors, with a specific focus on differences across work motivation dimensions (rooted in Self-Determination Theory, SDT).

**Methods:**

A multicenter cross-sectional design was adopted. Data from 701 clinical nurses across six hospitals in Guangxi Province were analyzed: LPA identified four distinct profiles, and Multinomial Logistic Regression was used to examine predictors. Work motivation was measured by the Multidimensional Work Motivation Scale (MWMS), and voice behavior by the Voice Behavior Scale (VBS).

**Results:**

LPA identified four distinct profiles (Conservative, 5.42%; Balanced Risk-Taker, 26.39%; Transitional, 34.38%; Challenging, 33.8%), and Multinomial Logistic Regression was used to examine predictors. Work motivation was measured by the Multidimensional Work Motivation Scale (MWMS), and voice behavior by the Voice Behavior Scale (VBS). Results showed autonomous motivation (e.g., intrinsic drive) strongly predicted active voice behavior, while amotivation predicted conservative profiles. Nurses exhibited high work motivation (MWMS: 93.02 ± 21.09) and moderately high voice behavior (VBS: 39.27 ± 8.736).

**Discussion:**

The research found that nurses exhibited high work motivation and moderately high voice behavior, with autonomous motivation being a pivotal predictor. Differentiated strategies targeting intrinsic motivation enhancement are critical for fostering nursing innovation and improving care quality.

## Highlights

- Nurses’ voice behavior can be categorized into four distinct profiles (Conservative, Balanced Risk-Taker, Transitional, and Challenging), revealing significant heterogeneity in their willingness to express opinions.
- Autonomous motivation (e.g., intrinsic drive and identified regulation) strongly promotes voice behavior, while amotivation and excessive reliance on material rewards may inhibit it.
- Tailored management strategies emphasizing intrinsic motivation, fair evaluation mechanisms, and differentiated support are critical to enhancing nurses’ voice behavior and overall care quality.

## Introduction

In today’s healthcare environment, nurses play a pivotal role in optimizing care delivery and patient satisfaction (Chen, 2019), with their voice behavior—defined as the voluntary expression of safety-related concerns or suggestions for organizational improvement (Van Dyne and LePine, 1998)—serving as a key driver of team effectiveness, nursing quality, and patient safety (Garon, 2012; Lee et al., 2022; Schwappach and Gehring, 2014; Zhou et al., 2021). Positive voice behavior lets nursing managers quickly spot clinical problems and hidden risks, boosting team performance, service quality, and cohesion (Guo et al., 2021). In contrast, inadequate voice behavior cuts efficiency, raises turnover intentions, and hides practice gaps (Atalla et al., 2022; Giesbers et al., 2016; Li et al., 2018).

However, the heterogeneous expression of nurses’ voice behavior across individuals has been largely neglected by traditional variable-centered methods, such as classifying voice into promotive versus prohibitive types based on total scores. These approaches fail to account for individual variability and are insufficient for identifying latent subgroups of voice behavior (Guo et al., 2021; Islam et al., 2019). Notably, traditional cut-off approaches [e.g., classifying voice behavior into “promotive” or “prohibitive” subtypes based on total scale scores, as employed in foundational research on voice behavior (Liang et al., 2012)] are inherently limited. These methods rely on the untested assumption that voice behavior follows linear and homogeneous patterns across individuals, implying a uniform expression of voice tendencies among all nurses (Hicks et al., 2017). In practice, however, individuals often exhibit complex and heterogeneous combinations of voice behaviors. For instance, a nurse may demonstrate moderate levels of both promotive and prohibitive voice, rather than exhibiting a high score on one dimension and a low score on the other; this constitutes a nuanced behavioral profile that conventional cut-off methods fail to capture (Muthén and Muthén, 2000). Specifically, Implicit Voice Theories (IVTs)—defined as taken-for-granted beliefs about the risk or inappropriateness of speaking up in hierarchical organizations (Şahin et al., 2021)—have been shown to predict defensive silence and constrain constructive voice, even in environments that formally encourage voice (Detert and Edmondson, 2011; Şahin et al., 2021). For instance, nurses holding beliefs such as “don’t embarrass the boss in public” or “voice leads to negative career consequences” may deliberately suppress opinions to avoid perceived risks (Şahin et al., 2021), a nuanced behavioral pattern that conventional variable-centered methods fail to capture. Variable-centered methods prioritize average relationships between variables, rather than identifying distinct subgroups of individuals, which can oversimplify behavioral complexity and limit the specificity of practical interventions (Hickendorff et al., 2018; McMullen and Hickendorff, 2018).

To address this gap, we employ Latent Profile Analysis (LPA), which is an individual-centered statistical method that identifies hidden subgroups via latent class variables (He and Fan, 2018; Woo et al., 2017). This approach is critical for unpacking the nuanced patterns of nurses’ voice behavior, which variable-centered methods cannot fully capture.

Work motivation is a key influencer of nurses’ voice behavior (Galletta et al., 2016; Bandhu et al., 2024), and we frame this investigation using Self-Determination Theory (SDT)—a macro-theory of human motivation that emphasizes motivation as a multidimensional system driven by the satisfaction of basic psychological needs (autonomy, competence, and relatedness) (Deci and Ryan, 1985, 2000, 2008). As the foundational proposition of SDT, autonomous motivation (encompassing intrinsic motivation and identified regulation) originates from individuals’ inherent psychological needs and value alignment, while controlled motivation (including amotivation, extrinsic regulation, and introjected regulation) is triggered by external pressures or rewards (Gagné et al., 2015; Ryan and Deci, 2020). This classification not only aligns with core SDT tenets (Deci and Ryan, 1985) but also gains cross-context support from Ozcan et al. (2023), who demonstrated that autonomous motivation fosters thriving—a psychological state integrating learning and vitality—and subsequent proactive behaviors (e.g., entrepreneurial intention), highlighting its generalizability to workplace contexts such as nursing. Consistently, autonomous motivation has been linked to proactive workplace behaviors (Ryan and Deci, 2020), while amotivation and excessive controlled motivation may constrain such behaviors by undermining psychological need satisfaction (Gagné and Deci, 2005).

Given the motivational challenges inherent in nursing practice (Atalla et al., 2022; Li et al., 2018), this study uses LPA to classify nurses’ voice behavior into latent profiles, then explores how SDT-based motivation dimensions and demographics predict profile membership. The goal is to provide nursing managers with targeted strategies to enhance voice behavior, thereby improving care quality and patient safety.

## Methods

### Participants

This study was conducted as a multicenter cross-sectional survey using a convenience sampling method. Between June and October 2024, a total of 790 clinical nurses from six hospitals of grade two or above were recruited for a questionnaire survey in Nanning, Guilin, and Qinzhou cities. The inclusion criteria for participants were as follows: (1) aged 18 years or older; (2) possessing a nurse qualification certificate and having worked for at least 1 year. The exclusion criteria were: (1) nurses in positions of head nurse or above; (2) nurses on rotation, those undergoing further training, or those on leave during the research period. Based on the principle of sample size estimation, the sample size should be 5 to 10 times the number of items. Given that this study comprised 82 items, and considering a 10% dropout rate, the minimum required sample size was calculated to be at least 457 cases.

This study was approved by the hospital ethics committee on January 3, 2025, with the ethics number: 2025-E0010. In this study, researchers clearly stated the voluntary nature and overall objectives on the first page of the questionnaire, emphasizing strict measures to maintain the confidentiality and anonymity of the participants. To protect personal identities, only numerical identifiers were used on the questionnaire, avoiding any data that could identify the nurses. Consequently, no specific information about any nurse will be disclosed in any publications. In compliance with Article 32 of the “Ethical Review Measures for Human Life Science and Medical Research” (National Health Commission of the People’s Republic of China, 2023), the requirement for written informed consent was waived because the research utilized anonymous data collection without physiological intervention, sensitive personal data, or commercial interests. Implied consent was obtained from participants by completing the questionnaire after they were presented with a cover page detailing the study.

### Data collection

All participants provided informed consent and voluntarily participated in this study. The study population was rigorously determined based on the inclusion and exclusion criteria. Data collection was facilitated online through the “Wenjuanxing” platform, which is a widely used survey tool in China that allows for the creation, distribution, and analysis of questionnaires in an efficient and secure manner. The platform uses end-to-end encryption for data transmission and storage; no personal identifiers (e.g., name, ID number) were collected (only numerical codes for tracking). To ensure the validity and scientific rigor of the questionnaire, participants were informed of the content and purpose of the survey, and assured that the survey was anonymous to obtain authentic information. The individual nurses themselves completed all items in the questionnaire. The principles for excluding invalid questionnaires were as follows:

1. Based on the time range observed during a pre-survey, questionnaires completed in less than the minimum response time (150 s) were excluded from the formal survey.
2. Questionnaires exhibiting obvious patterns or inconsistencies were also excluded.

In total, 790 questionnaires were collected, of which 89 were deemed invalid, resulting in 701 valid questionnaires with an effective response rate of 88.73%.

### Measures

The scales used in this study were professionally translated to Chinese.

#### Demographic characteristics questionnaire

Collected data included gender, age, educational background, professional title, employment type, hospital type, years of work experience, whether the participant is an only child, marital status, average monthly income, whether the participant is a specialized nurse, department, involvement in department management, participation in teaching tasks within the department, and gender of the head nurse. A total of 15 items were included.

#### Multidimensional work motivation scale (MWMS)

The MWMS, refined by Gagné et al. (2015), is an assessment tool based on the multidimensional concept of motivation within Self-Determination Theory (SDT). It is used to measure types of employee work motivation. This scale has been validated across multiple countries and languages, making it suitable for assessing work motivation in cross-cultural contexts. It comprises six dimensions: Amotivation, External Regulation—Social, External Regulation—Material, Intrinsic Motivation, Identified Regulation, and Introjected Regulation, with a total of 19 items. A Likert 7-point rating scale (ranging from 1 to 7, corresponding to “completely disagree” to “completely agree”) is employed. The total score ranges from 19 to 133, with higher scores indicating stronger motivation toward a specific type. The Cronbach’s *α* coefficient of this scale demonstrates high internal consistency across different subscales and language versions. In the original study, most subscales had Cronbach’s *α* coefficients >0.70, and in this study, the Cronbach’s α coefficient for the overall scale was 0.928, indicating high internal consistency.

#### Voice behavior scale (VBS)

The Voice Behavior Scale, designed by Liang et al. (2012), is an assessment tool aimed at measuring employees’ levels of voice behavior within an organization. It consists of two dimensions: proactive voice behavior and prohibitive voice behavior, with a total of 10 items. Each item uses a Likert 5-point rating scale, ranging from “strongly disagree” to “strongly agree,” corresponding to scores of 1 to 5, respectively. The total score of the scale ranges from 10 to 50, with higher scores reflecting more positive voice behavior among employees. The Cronbach’s *α* coefficient of this scale is 0.915, indicating good internal consistency. In this study, the Cronbach’s α coefficient for this scale was 0.962.

#### Pretest

Prior to the commencement of the study, a pretest was conducted among 30 randomly selected participants who met the inclusion and exclusion criteria from the aforementioned six hospitals. During the pretest, the research team closely monitored participants’ feedback and recorded the time taken to complete the questionnaire, as well as any questions or suggestions they had regarding various sections of the questionnaire. Based on their suggestions and confusion, revisions and improvements were made to the questionnaire. In the pretest, the Cronbach’s α coefficient for the Nurse Voice Behavior Scale was 0.929, and the Cronbach’s α coefficient for the Multidimensional Work Motivation Scale (MWMS) was 0.774.

#### Data analysis

The data collected through an online survey platform^1^ were exported to an Excel spreadsheet and verified by two researchers independently. Statistical analysis was conducted using SPSS 26.0 software. For continuous variables, the mean ± standard deviation was used for description, while for categorical variables, frequency, composition ratio, and rates were employed. Chi-square tests or rank-sum tests were applied for comparisons between groups. Analysis of Variance (ANOVA) was used for normally distributed data, while non-parametric tests were utilized for skewed distributions.

Confirmatory factor analysis (CFA) of the MWMS was conducted using Mplus 8.3 software. Competitive models, including a six-factor model, a five-factor model, and a three-factor model, were constructed to assess the goodness-of-fit. The overall fit of the models was reflected by the ratio of the chi-square value (*Χ*^2^) to the degrees of freedom (df). The Comparative Fit Index (CFI) and Tucker–Lewis Index (TLI), serving as incremental fit indices, indicated better model fit as their values approached 1. A smaller Standardized Root Mean Square Residual (SRMR) was preferred, generally considered good if less than 0.08. Similarly, a smaller Root Mean Square Error of Approximation (RMSEA) was better, with values between 0.05 and 0.08 indicating a good fit and values less than 0.05 indicating an excellent fit.

Latent Profile Analysis (LPA) of nurses’ voice behavior was conducted in Mplus 8.3, starting with a baseline model of a single class and gradually increasing the number of classes. Model selection was based on the Akaike Information Criteria (AIC), Bayesian Information Criteria (BIC), and adjusted Bayesian Information Criteria (aBIC), with lower values indicating better model fit. An entropy value closer to 1 indicated higher classification accuracy. The Lo–Mendell–Rubin (LMR) test and the Bootstrap Likelihood Ratio Test (BLRT) were used to assess differences in fit between models, with a *p*-value less than 0.05 indicating that a model with an increased number of classes was significantly better than the previous one. When determining the optimal model, the practical significance of the classification was also considered.

Using the classification results from LPA as the dependent variable, multivariate Logistic Regression Analysis was conducted on variables that were statistically significant in the univariate analysis.

Given that all data in this study were self-reported and collected through the same online platform “Wenjuanxing,” there is a potential risk of common method bias (CMB). To assess this potential concern, we performed Harman’s single-factor test. The results showed that the first factor accounted for 51.272% of the total variance, which is well below the 60% threshold recommended by Podsakoff et al. (2003). This indicates that common method bias is not a serious issue in the present study.

## Results

### General characteristics of study participants

A total of 701 clinical nurses were included in this study. The general characteristics of the participants are presented in Table 1.

**Table 1:** General characteristics of study participants (*N* = 701).

Data type		Items	Frequency (n)	Percentage (%)
Demographics	Gender	Male	40	5.7
		Female	661	94.3
	Age/years	18 ~ 25	156	22.3
		26 ~ 30	156	22.3
		31 ~ 40	281	40.1
		41 ~ 50	92	13.1
		>51	16	2.3
	Educational background	Junior college degree or below	96	13.7
		Undergraduate (including post-secondary)	597	85.2
		Postgraduate and above	8	1.1
	Whether the participant is an only child	Yes	98	14.0
		No	603	86.0
	Marital status	Unmarried	270	38.5
		Married	411	58.6
		Divorced	20	2.9
Work-related characteristics	Professional title	Nurse	136	19.4
		Nurse Practitioner	253	36.1
		Charge Nurse	241	34.4
		Deputy Director of Nursing	64	9.1
		Director of Nursing	7	1.0
	Mode of employment	Establishment	132	18.8
		Non-tenured	569	81.2
	Type of hospital	Tertiary hospitals	631	90.0
		Secondary hospital or below	70	10.0
	Years of work experience/years	<5	207	29.5
		5 ≤ Y<10	157	22.4
		10 ≤ Y<15	180	25.7
		15 ≤ Y<20	72	10.3
		≥20	85	12.1
	Average monthly income/RMB (yuan)	≤4,000	58	8.3
		4,001 ~ 7,000	311	44.4
		7,001 ~ 10,000	239	34.1
		≥10,000	93	13.3
	Whether the participant is a specialized nurse	Yes	157	22.4
		No	544	77.6
	Department	Internal Medicine	256	36.5
		Surgery	179	25.5
		Obstetrics and Gynecology	39	5.6
		Pediatrics	34	4.9
		Emergency	12	1.7
		ICU	80	11.4
		Operating theater	16	2.3
		Outpatient	6	0.9
		Others	79	11.3
	Whether the involvement in department management	Yes	310	44.2
		No	391	55.8
	Whether the participation in teaching tasks within the department	Yes	343	48.9
		No	358	51.1
	Gender of the head nurse	Male	12	1.7
		Female	689	98.3

### Latent profile analysis of nurses’ voice behavior

In this study, a latent profile model was constructed based on the scores of 10 items from the nurses’ voice behavior scale. Models with 1–5 latent classes were fitted, with the specific fit indices presented in Table 2. As the number of latent classes increased, the AIC, BIC, and aBIC indices exhibited a decreasing trend, indicating an increase in model complexity. The entropy values of all models (from 2 to 5 classes) were above 0.800, demonstrating high classification accuracy. Additionally, the LMR and BLRT indices (both *p* < 0.001) for models with 2 to 4 classes were significant, suggesting that adding a class improved model fit; however, the LMR *p*-value for the 5-class model was 0.136 (not significant), indicating that the 5-class model did not provide a better fit than the 4-class model. While the downward trend of AIC/BIC slowed only slightly when increasing from 4 to 5 classes (as observed in visual analysis), we further evaluated the 5-class model from theoretical and interpretability perspectives: the 5-class model generated a very small profile (only 1.85% of the sample), which lacked sufficient theoretical/clinical relevance to nurses’ voice behavior. Considering both the simplicity and interpretability of the model, the 4-latent profile model was ultimately selected as the best-fitting model in this study.

**Table 2:** Fit indices for latent profile models of nurses’ voice behavior items.

Profile	Log likelihood	AIC	BIC	aBIC	*p*-value		Entropy	Profile prevalence (%)
					LMR	BLRT		
1	−10011.810	20063.621	20154.671	20091.167				
2	−8075.948	16213.896	16355.024	16256.593	0.000	0.000	0.943	253(36.09%)/448(63.91%)
3	−7209.634	14503.267	14694.473	14561.114	0.000	0.000	0.976	41(5.85%)/281(40.09%)/379(54.07%)
4	−6454.701	13015.403	13256.685	13088.4	0.001	0.000	0.976	185(26.39%)/38(5.42%)/241(34.38%)/237(33.8%)
5	−6293.418	12714.836	13006.196	12802.983	0.139	0.000	0.979	13(1.85%)/28(3.99%)/183(26.11%)/239(34.09%)/238(33.95%)

AIC, Akaike Information Criterion; BIC, Bayesian Information Criterion; aBIC, adjusted BIC; LMR, Lo–Mendell–Rubin test; BLRT, Bootstrap Likelihood Ratio Test.

**Figure 1:**
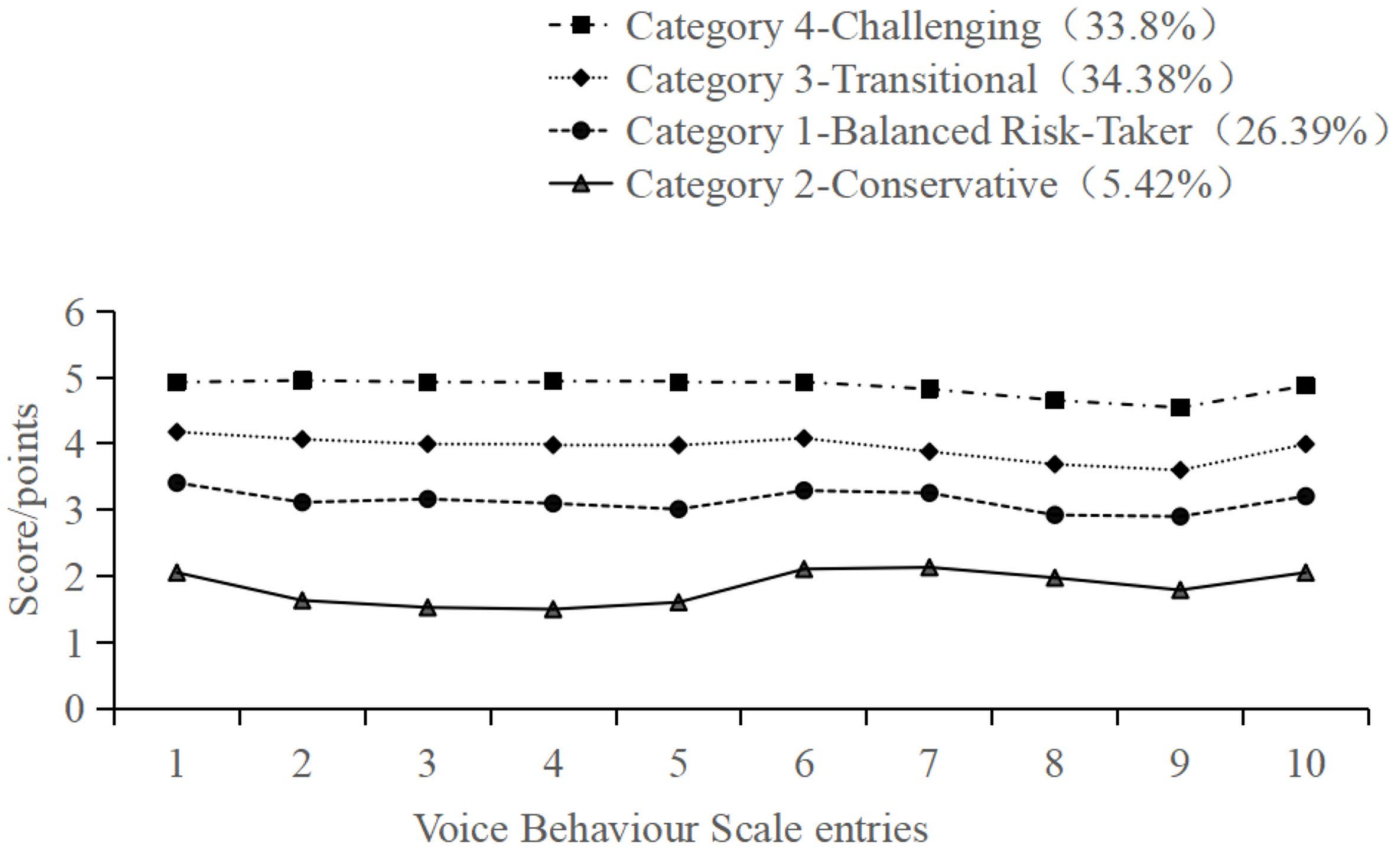
Characteristic distribution of four latent classes of nurses’ voice behavior.

Based on the scoring characteristics of the four latent profiles across the 10 items of the voice behavior scale (Figure 1), each latent profile exhibited consistent trends without intersections in item scores. Drawing on the research by Xiao et al. (2024), the four categories were named as follows: Category 2, consisting of 38 nurses (5.42%) and representing the smallest proportion, had the lowest scores on the voice behavior items, particularly in the dimension of promotive voice. These nurses were named the “Conservative.” Category 1, comprising 185 nurses (26.39%), scored in the lower-middle range across items and could be termed the “Balanced Risk-Taker.” Category 3, with the largest proportion of 241 nurses (34.38%), scored in the upper-middle range across items but had lower scores in the dimension of prohibitive voice. These nurses were designated the “Transitional.” Category 4, including 237 nurses (33.8%), had the highest item scores with the smallest differences among items, earning them the label of the “Challenging.”

### Scores on the voice behavior scale (VBS) and the MWMS of the study participants

The scores obtained on the scales in this survey are presented in Table 3.

**Table 3:** Scores on the voice behavior scale (VBS) and the multidimensional work motivation scale MWMS of the study participants.

Scale	Items (n)	Score (mean ± SD)
Nurse’ Voice Behavior	10	(39.27 ± 8.736)
Promotive Voice Behavior	5	(19.35 ± 4.515)
Prohibitive Voice Behavior	5	(19.92 ± 4.614)
Work Motivation	19	(93.02 ± 21.09)
Amotivation	3	(6.43 ± 5.07)
Extrinsic Regulation—Social	3	(14.64 ± 5.674)
Extrinsic Regulation—Material	3	(17.03 ± 4.015)
Introjected Regulation	4	(21.78 ± 5.869)
Identified Regulation	3	(17.42 ± 4.148)
Intrinsic Motivation	3	(15.72 ± 4.992)

### Confirmatory factor analysis (CFA) of the MWMS

The internal consistency reliability of various dimensions of the MWMS was analyzed, with Cronbach’s *α* coefficients all exceeding 0.8 (and notably, all greater than 0.7), meeting the prerequisite for factor analysis, as shown in Table 4. In this paper, CFA was conducted separately on the three-factor model based on Self-Determination Theory (SDT), the five-factor model of the MWMS, and a six-factor model that subdivides extrinsic regulatory motivation into social and material dimensions. The results are presented in Table 5, with the six-factor model demonstrating the best goodness-of-fit.

**Table 4:** Internal consistency reliability of the MWMS and its dimensions.

Name	Corresponding items	Cronbach’s *α*	KMO and Bartlett’s test
Multidimensional Work Motivation Scale	19	0.928	0.918
Amotivation	3	0.961	0.768
Extrinsic Regulation—Social	3	0.926	0.747
Extrinsic Regulation—Material	3	0.89	0.705
Introjected Regulation	4	0.915	0.748
Identified Regulation	3	0.97	0.773
Intrinsic Motivation	3	0.977	0.784

MWMS, Multidimensional Work Motivation Scale.

**Table 5:** Results of confirmatory factor analysis.

Model	*x* ^2^	df	CFI	TLI	SRMR	RMSEA
Six-factor Model	618.092	137	0.936	0.92	0.035	0.071
Five-factor Model	1331.002	142	0.845	0.814	0.064	0.108
Three-factor Model	2271.779	149	0.719	0.677	0.076	0.143

The six-factor model includes Amotivation, Extrinsic Regulation—Social, Extrinsic Regulation—Material, Intrinsic Motivation, Identified Regulation, and Introjected Regulation; the five-factor model combines Extrinsic Regulation into a single factor encompassing both social and material aspects, alongside Amotivation, Intrinsic Motivation, Identified Regulation, and Introjected Regulation; the three-factor model comprises Autonomous Motivation (including Intrinsic Motivation and Identified Regulation), Controlled Motivation (including Extrinsic Regulation—Social, Material, and Introjected Regulation), and Amotivation.

### Univariate analysis of latent profiles of nurses’ voice behavior

Based on the latent profile analysis of nurses’ voice behavior, a univariate analysis was conducted on the collected general data. The results are shown in Table 6.

**Table 6:** Univariate analysis of general data and different types of voice behavior latent profiles among nurses (*N* = 701).

	Items	“Conservative” (*n* = 38)	“Balanced Risk-Taker” (*n* = 185)	“Transitional” (*n* = 241)	“Challenging” (*n* = 237)	*X*^2^-value	*p*-value
Gender	Male	2(5.3)	8(4.3)	17(7.1)	13(5.5)	1.453	0.691
	Female	36(94.7)	177(95.7)	224(92.9)	224(94.5)		
Age/years	18 ~ 25	14(36.8)	57(30.8)	56(23.2)	29(12.2)	54.319	<0.001
	26 ~ 30	10(26.3)	50(27)	59(24.5)	37(15.6)		
	31 ~ 40	10(26.3)	64(34.6)	85(35.3)	122(51.5)		
	41 ~ 50	3(7.9)	11(5.9)	37(15.4)	41(17.3)		
	>51	1(2.6)	3(1.6)	4(1.7)	8(3.4)		
Educational background	Junior college degree or below	11(28.9)	21(11.4)	26(10.8)	38(16)	11.078	0.063
	Undergraduate (including post-secondary)	27(71.1)	163(88.1)	211(87.6)	196(82.7)		
	Postgraduate and above	0(0)	1(0.5)	4(1.7)	3(1.3)		
Professional title	Nurse	11(28.9)	48(25.9)	46(19.1)	31(13.1)		0.00277
	Nurse Practitioner	16(42.1)	73(39.5)	87(36.1)	77(32.5)		
	Charge Nurse	10(26.3)	53(28.6)	77(32)	101(42.6)		
	Deputy Director of Nursing	1(2.6)	11(5.9)	27(11.2)	25(10.5)		
	Director of Nursing	0(0)	0(0)	4(1.7)	3(1.3)		
Mode of employment	Establishment	7(18.4)	21(11.4)	54(22.4)	50(21.1)	9.588	0.022
	Non-tenured	31(81.6)	164(88.6)	187(77.6)	187(78.9)		
Type of hospital	Tertiary hospitals	32(84.2)	168(90.8)	217(90)	214(90.3)	1.728	0.635
	Secondary hospital or below	6(15.8)	17(9.2)	24(10)	23(9.7)		
Years of work experience/years	<5	16(42.1)	76(41.1)	73(30.3)	42(17.7)	43.312	<0.001
	5 ≤ Y<10	10(26.3)	45(24.3)	52(21.6)	50(21.1)		
	10 ≤ Y<15	6(15.8)	40(21.6)	58(24.1)	76(32.1)		
	15 ≤ Y<20	4(10.5)	12(6.5)	26(10.8)	30(12.7)		
	≥20	2(5.3)	12(6.5)	32(13.3)	39(16.5)		
Whether the participant is an only child	Yes	10(26.3)	28(15.1)	27(11.2)	33(13.9)	6.56	0.086
	No	28(73.7)	157(84.9)	214(88.8)	204(86.1)		
Marital status	Unmarried	20(52.6)	100(54.1)	94(39)	56(23.6)	46.792	<0.001
	Married	17(44.7)	84(45.4)	138(57.3)	172(72.6)		
	Divorced	1(2.6)	1(0.5)	9(3.7)	9(3.8)		
Average monthly income/RMB (yuan)	≤4,000	4(10.5)	13(7)	23(9.5)	18(7.6)	7.494	0.586
	4,001 ~ 7,000	20(52.6)	86(46.5)	111(46.1)	94(39.7)		
	7,001 ~ 10,000	10(26.3)	66(35.7)	76(31.5)	87(36.7)		
	≥10,000	4(10.5)	20(10.8)	31(12.9)	38(16)		
Whether the participant is a specialized nurse	Yes	9(23.7)	33(17.8)	55(22.8)	60(25.3)	3.436	0.33
	No	29(76.3)	152(82.2)	186(77.2)	177(74.7)		
Department	Internal Medicine	16(42.1)	69(37.3)	85(35.3)	86(36.3)		0.1259
	Surgery	3(7.9)	44(23.8)	63(26.1)	69(29.1)		
	Obstetrics and Gynecology	1(2.6)	12(6.5)	14(5.8)	12(5.1)		
	Pediatrics	1(2.6)	8(4.3)	17(7.1)	8(3.4)		
	Emergency	0(0)	7(3.8)	4(1.7)	1(0.4)		
	ICU	6(15.8)	22(11.9)	28(11.6)	24(10.1)		
	Operating theater	3(7.9)	4(2.2)	4(1.7)	5(2.1)		
	Outpatient	0(0)	0(0)	2(0.8)	4(1.7)		
	Others	8(21.1)	19(10.3)	24(10)	28(11.8)		
Whether the involvement in department management	Yes	6(15.8)	52(28.1)	112(46.5)	140(59.1)	53.612	<0.001
	No	32(84.2)	133(71.9)	129(53.5)	97(40.9)		
Whether the participation in teaching tasks within the department	Yes	9(23.7)	68(36.8)	122(50.6)	144(60.8)	34.211	<0.001
	No	29(76.3)	117(63.2)	119(49.4)	93(39.2)		
Gender of the head nurse	Male	1(2.6)	4(2.2)	2(0.8)	5(2.1)	2.397	0.463
	Female	37(97.4)	181(97.8)	239(99.2)	232(97.9)		

The study results revealed that nurses categorized as “Conservative” scored (64.18 ± 24.781) on the MWMS, “Balanced Risk-Takers” scored (80.28 ± 16.833), “Transitional” nurses scored (93.03 ± 15.439), and “Challenging” nurses scored (107.57 ± 16.911). Comparisons among the four groups showed statistically significant differences (Welch’s *F* = 81.612, *p* < 0.001). The total score of the MWMS and its six dimensions exhibited skewed distributions. The results of the Kruskal–Wallis *H* test, as presented in Table 7, demonstrated significant differences in the total score of the MWMS and its six dimensions across the four latent categories of nurses’ voice behavior (all *p* < 0.001). Comparisons of different profile types of nurses’ voice behavior in terms of age, professional title, employment type, years of work experience, marital status, whether assisting in department management, and whether having teaching responsibilities also showed statistically significant differences (all *p* < 0.05).

**Table 7:** Relationship between the MWMS scores and its six dimensions with nurses’ voice behavior.

Items	*H*-value	*p*-value
The total score of the MWMS	257.659	<0.001
Amotivation	58.89	<0.001
Extrinsic Regulation—Social	136.167	<0.001
Extrinsic Regulation—Material	218.355	<0.001
Introjected Regulation	227.034	<0.001
Identified Regulation	298.531	<0.001
Intrinsic Motivation	276.509	<0.001

### Multinomial logistic regression analysis of latent profiles of nurses’ voice behavior

We employed the four categories of nurses’ voice behavior as the dependent variable, and incorporated statistically significant indicators from univariate analyses as predictors in a multinomial logistic regression analysis. For this model, we specified the construction steps explicitly: all significant predictors identified in univariate analyses were entered simultaneously into the model in one step. Prior to regression, we performed collinearity diagnostics for all candidate variables. After removing the redundant total work motivation score variable, the variance inflation factor (VIF) values for all remaining variables were below 10, indicating no severe multicollinearity issues (Hair et al., 2010). The results indicated that the likelihood ratio χ^2^ of the model was 439.426, with a *p*-value < 0.001. Assisting in department management, Extrinsic Regulation-Material, Identified Regulation, and Intrinsic Motivation were identified as factors influencing the latent categories of nurses’ voice behavior (*p* < 0.001 or *p* < 0.05). The assignment of independent variables is shown in Table 8.

**Table 8:** Assignment of independent variables for latent profiles of nurses’ voice behavior.

Variable	Assignment method
Age/years	18 ~ 25 = 1, 26 ~ 30 = 2, 31 ~ 40 = 3, 41 ~ 50 = 4, >51 = 5
Professional Title	Nurse = 1, Nurse Practitioner = 2, Charge Nurse = 3, Deputy Director of Nursing = 4, Director of Nursing = 5
Years of Work Experience/years	<5 = 1, 5 ≤ Y<10 = 2, 10 ≤ Y<15 = 3, 15 ≤ Y<20 = 4, ≥20 = 5
Marital Status	Unmarried = 1, Married = 2, Divorced = 3
Assistance in Department Management	Yes = 1, No = 2
Teaching Assignment	Yes = 1, No = 2
Scores for Each Dimension of the MWMS	Log-transformed original values

In the model incorporating scores of work motivation dimensions: For the comparison between C1 (Conservative Type) and C2 (Balanced Risk-Taker Type): Nurses with higher scores in Amotivation and Intrinsic Motivation, as well as unmarried nurses, were more likely to be classified into C2. For comparisons between C1 and C3 (Transitional Type), and between C1 and C4 (Challenging Type): Nurses with the title of “staff nurse” and higher Amotivation scores were more likely to remain in C1; Nurses involved in department management, or with higher scores in Extrinsic Regulation-Material, Identified Regulation, and Intrinsic Motivation, were more likely to belong to C3 or C4; Additionally, nurses with teaching responsibilities were more likely to be categorized into C3. For comparisons between C2 and C3, and between C2 and C4: Nurses with the title of “staff nurse” were more likely to remain in C2; Nurses with higher scores in Extrinsic Regulation-Material and Intrinsic Motivation or involved in department management were more likely to belong to C3 or C4; Those with higher Identified Regulation scores were more likely to be classified into C4. For the comparison between C3 and C4: Nurses with higher scores in Identified Regulation and Intrinsic Motivation were more likely to belong to C4. See Table 9 for detailed results.

**Table 9:** Results of multinomial logistic regression analysis for latent profiles of nurses’ voice behavior (x = scores on dimensions of the MWMS).

Comparison and variable	Regression coefficient	Standard error	Wald *x*^2^ value	*p*-value	OR	95%CI
C1 vs. C2^1^						
Amotivation	0.817	0.368	4.922	0.027	2.264	1.100 ~ 4.659
Intrinsic Motivation	1.256	0.569	4.878	0.027	3.512	1.152 ~ 10.708
Marital status-Unmarried	3.604	1.612	4.996	0.025	36.749	1.558 ~ 866.576
C1 vs. C3^1^						
Professional title-Nurse	−13.337	1.907	48.893	<0.001	1.61 × 10^−6^	3.840 × 10^−8^ ~ 6.782 × 10^−5^
Assisting Management-Yes	1.737	0.605	8.233	0.004	5.68	1.734 ~ 18.607
Whether the participation in teaching tasks within the department-Yes	1.199	0.601	3.988	0.046	3.318	1.022 ~ 10.769
Amotivation	−2.828	0.676	17.521	<0.001	0.059	0.016 ~ 0.222
Extrinsic Regulation—Material	1.955	0.779	6.308	0.012	7.067	1.536 ~ 32.504
Identified Regulation	2.398	0.952	6.337	0.012	10.997	1.700 ~ 71.114
Intrinsic Motivation	2.131	0.623	11.683	<0.001	8.422	2.482 ~ 28.579
C1 vs. C4^1^						
Professional title-Nurse	−12.555	1.764	50.643	<0.001	3.527 × 10^−6^	1.111 × 10^−7^ ~ 0.000
Assisting Management-Yes	2.100	0.629	11.131	<0.001	8.167	2.378 ~ 28.043
Extrinsic Regulation—Material	1.901	0.842	5.103	0.024	6.696	1.286 ~ 34.858
Identified Regulation	9.837	1.510	42.416	<0.001	18716.870	969.466 ~ 361354.668
Intrinsic Motivation	3.171	0.749	17.929	<0.001	23.843	5.493 ~ 103.495
C2 vs. C3^2^						
Professional title-Nurse	−15.905	1.103	207.811	<0.001	1.237 × 10^−7^	1.423 × 10^−8^ ~ 1.076 × 10^−6^
Assisting Management-Yes	0.671	0.256	6.852	0.009	1.956	1.184 ~ 3.233
Extrinsic Regulation—Material	1.486	0.536	7.688	0.006	4.421	1.546 ~ 12.642
Intrinsic Motivation	0.875	0.388	5.082	0.024	2.398	1.121 ~ 5.129
C2 vs. C4^2^						
Professional title-Nurse	−15.123	0.817	342.838	<0.001	2.705 × 10^−7^	5.456 × 10^−8^ ~ 1.341 × 10^−6^
Assisting Management-Yes	1.034	0.300	11.910	<0.001	2.812	1.563 ~ 5.059
Extrinsic Regulation—Material	1.432	0.607	5.565	0.018	4.189	1.274 ~ 13.770
Identified Regulation	8.382	1.336	39.339	<0.001	4368.099	318.230 ~ 59957.486
Intrinsic Motivation	1.915	0.559	11.723	<0.001	6.789	2.268 ~ 20.320
C3 vs. C4^3^						
Identified Regulation	7.523	1.299	33.528	<0.001	1849.728	144.953 ~ 23604.209
Intrinsic Motivation	1.041	0.528	3.887	0.049	2.831	1.006 ~ 7.966

C1 = Conservative Type, C2 = Balanced Risk-Taker Type, C3 = Transitional Type, C4 = Challenging Type.

^1^C1 was used as the reference group. ^2^C2 was used as the reference group. ^3^C3 was used as the reference group.

## Discussion

### Four latent profile types of nurses’ voice behavior

Latent Profile Analysis (LPA) identified profiles of nurse voice behavior: “Conservative,” “Balanced Risk-Taker,” “Transitional” and “Challenging.” This nuanced classification extends beyond prior three-profile models (Xiao et al., 2024) by capturing greater heterogeneity in how nurses express concerns and suggestions. However, as a cross-sectional study, the observed relationships are associational; causal directions cannot be established.

#### Conservative type (C1): “status maintenanceers” under motivation suppression

Within the scope of this study, the core characteristics of “Conservative” nurses were young (<30 years old), junior professional title, non-establishment (Unstable employment status), working experience < 10 years, unmarried, not involved in department management and not undertaking teaching tasks (Table 1). This result can be interpreted through three interconnected lenses: (1) Motivational suppression: Amotivation directly undermines the internal drive for voice behavior, aligning with SDT’s core principle that amotivation inhibits proactive behaviors (Deci and Ryan, 2008). (2) Implicit voice theories: Consistent with Şahin et al. (2021), Conservative nurses strongly endorsed beliefs such as “negative career consequences of voice” and “don’t embarrass the boss in public”, which foster defensive silence to avoid perceived risks. In Guangxi’s hierarchical medical environment, junior nurses may internalize these beliefs to maintain occupational safety, as IVTs often operate as taken-for-granted norms in collectivistic cultures (Şahin et al., 2021). (3) Occupational insecurity: Non-tenured status and limited work experience reduce perceived job security, reinforcing a risk-averse attitude toward voice (Li and Song, 2024).

#### Balanced risk-taker type (C2): “prudent decision-maker” under motivational contradictions

The “Balanced Risk-Taker” profile shares similar demographic characteristics with the “Conservative” profile (i.e., younger age, junior professional title, non-tenured status, less than 15 years of work experience, unmarried, and no involvement in management or teaching responsibilities) (Table 1). However, regression results revealed that these nurses were more distinguishable from the “Conservative” (C1) profile due to their simultaneously higher scores in both amotivation and intrinsic motivation, along with being unmarried. This “motivational conflict” reflects the professional dilemma faced by early-career nurses: on the one hand, intrinsic motivation fosters a degree of enthusiasm and willingness to improve their work; on the other hand, amotivation (potentially stemming from occupational stress or uncertainty about career development) suppresses their initiative to speak up, ultimately resulting in a behavioral pattern characterized as “weighing risks and benefits while voicing opinions prudently.” Compared to the “Conservative” profile, these nurses possess slightly more work experience and a better capacity for risk assessment, enabling them to seek a balance between “maintaining the “advocating for minor changes.” The existence of this subgroup further demonstrates the heterogeneity in nurses’ voice behavior, suggesting that traditional binary classification approaches are inadequate for capturing such intermediate profiles.

#### Transition type (C3): the largest group, “conservative reformers”

In this study, “Transitional” nurses accounted for the highest proportion (34.38%), with core characteristics including older age (>30 years old), intermediate professional title, permanent employment status within China’s healthcare system (i.e., stable and formal employment), work experience exceeding 5 years, and assuming preceptorship responsibilities without participating in departmental management (Table 1). Regression results indicated that these nurses were more likely to transition from “conservative-type” to “active-type” due to higher scores in external regulation-material, identified regulation, and intrinsic motivation, as well as undertaking preceptorship tasks and assisting in departmental management. This finding aligns with Karataş and Çankır (2023), who emphasized that collegial solidarity and administrative support—key contextual factors for transitional nurses—mediate the relationship between individual motivation and work-related proactive behaviors. Specifically, the preceptorship role endows them with the identity of knowledge transmitters, prompting attention to nursing process optimization (Chen et al., 2012). Additionally, the mediating role of thriving at work (a construct integrating learning and vitality; Porath et al., 2012) may further explain this transition, as autonomous motivation fosters thriving, which in turn promotes constructive voice (Ozcan et al., 2023; Karataş and Çankır, 2023). Meanwhile, the long-term career stability brought by permanent employment status (i.e., “perceived job security from stable employment” in international research) further reduces the perceived risk of voice behavior (Falatah et al., 2021). Combined with the regional cultural characteristics of Guangxi, these nurses, influenced by collectivist culture, emphasize team stability and also possess a certain willingness for change due to professional maturity, thus forming the voice characteristic of “steady transition.” This finding also suggests that in collectivist-oriented healthcare settings, “intermediate-type” voice actors may serve as key bridges for promoting organizational change.

#### Challenging type (C4): autonomous motivation-driven “change leader”

The “Challenging” profile represents the most change-oriented group of nurses, characterized by seniority (over 15 years of work experience), intermediate or senior professional titles, tenured status, being married, and involvement in both departmental management and clinical teaching. Regression results further clarified that nurses with higher scores in identified regulation and intrinsic motivation, and those involved in departmental management, were more likely to belong to this profile. When compared to the “Transitional” (C3) profile, “Challenging” nurses exhibited significantly higher levels of both identified regulation and intrinsic motivation. This finding strongly supports the core assumption of Self-Determination Theory (SDT) that autonomous motivation, which encompasses both intrinsic motivation and identified regulation, serves as a key driver prompting individuals to engage in proactive voice behavior (Ryan and Deci, 2020; Chen et al., 2024). The professional confidence gained from seniority, the sense of security associated with tenured positions, and the voice afforded by participation in management collectively reinforce their willingness to challenge the status quo through their voice behavior. From a managerial perspective, the presence of such nurses injects innovative vitality into organizations, and their development is closely linked to the presence of a supportive environment for voice behavior within the organization, exemplified by access to management participation channels and opportunities for career development.

### The overall level of nurses’ voice behavior and work motivation

The results of this study indicated that nurses’ voice behavior was generally at a moderately high level (total score of VBS: 39.27 ± 8.736), and their overall work motivation scored relatively high (total score of MWMS: 93.02 ± 21.09), which is consistent with previous research findings (Guo et al., 2021; Islam et al., 2019; Xiao et al., 2024). At the dimensional level, the score of promotive voice was slightly lower than that of prohibitive voice. This may be associated with the hierarchical culture of the healthcare environment in Guangxi, as nurses—especially those with higher professional titles, who account for 44.51% of the sample—are more inclined to maintain team harmony and patient safety through cautious prohibitive voice, avoiding potential conflicts arising from innovative suggestions (Zhang et al., 2024). Among the dimensions of work motivation, intrinsic motivation scored the lowest while identified regulation scored the highest, suggesting that nurses’ work motivation stems more from professional identity and self-actualization (Ayalew et al., 2021; Baljoon et al., 2018) rather than mere work enjoyment.

### 1.1 The correlations between the various dimensions of work motivation and nurses’ suggestion-giving behavior

Building on the latent profile analysis, this study delves into the predictive roles of work motivation dimensions derived from Self-Determination Theory (SDT), which serve as the core explanatory mechanism for the heterogeneity in nurses’ voice behavior. The findings move descriptive classifications to reveal how motivational drivers differentially influence profile membership, offering nuanced insights for targeted interventions.

#### Autonomous motivation: the core driving factor of the act of offering suggestions

Autonomous motivation—comprising intrinsic motivation and identified regulation—emerged as the most potent predictor of active voice behavior. Nurses with higher scores in these dimensions were significantly more likely to belong to the Transitional (C3) and Challenging (C4) profiles. The profound predictive power of identified regulation for the Challenging profile underscores that when nurses internalize the value of their work, voice behavior transforms from discretionary participation to a professional obligation aligned with their self-concept (Ryan and Deci, 2020). This mechanism is consistent with Ozcan et al. (2023), who found that autonomous motivation (especially identified regulation) enhances thriving by satisfying psychological needs for autonomy and competence, thereby fostering sustained proactive behaviors. In the context of nursing, challenging-type nurses’ high identified regulation reflects their alignment of voice behavior with professional ethics and patient safety goals, while their high intrinsic motivation fuels continuous learning and innovation in care delivery (Ozcan et al., 2023). Together, these motivational processes create a virtuous cycle: autonomous motivation promotes thriving, which in turn strengthens constructive voice (Karataş and Çankır, 2023). This aligns with SDT’s tenet that autonomous motivation fulfills psychological needs for autonomy and competence, thereby fostering proactive and sustained behaviors (Gagné et al., 2015). However, the cross-sectional design necessitates caution against causality; longitudinal studies are needed to explore whether autonomous motivation drives voice or vice versa.

To effectively enhance the sense of autonomy, the strategies we can adopt include: providing diversified career development opportunities (Kohnen et al., 2023), increasing the interest and challenge of work content (Bogdán et al., 2024), and creating a positive and supportive work environment (Aleo et al., 2024). These targeted initiatives are expected to motivate nurses to more proactively engage in nursing work, thereby driving an overall improvement in nursing quality and work efficiency (Zeng et al., 2022).

#### Amotivation: a dual pathway to voice inhibition

Amotivation consistently predicted membership in conservative profiles (C1 and C2). Its positive association with the Balanced Risk-Taker profile (C2)—despite this group’s motivation—reveals a “motivational conflict.” This paradox reflects early-career nurses’ dilemma: intrinsic interest is stifled by uncertainties in career development or perceived organizational indifference, leading to a cautious, risk-weighing approach to voice (Li and Song, 2024). For Conservative nurses (C1), amotivation directly undermines the internal drive for voice, aligning with SDT’s emphasis on amotivation as a barrier to proactive behavior (Deci and Ryan, 2008).

However, in interpreting the motivational conflict specific to the Balanced Risk-Taker profile (C2, *n* = 38, 5.42%), caution is warranted due to its relatively smaller sample size compared to other profiles. While the observed association is statistically significant, its magnitude and generalizability should be viewed as preliminary. Future research with larger, balanced samples is needed to confirm the robustness of this nuanced motivational dynamic.

To address amotivation, establishing a fair and transparent evaluation mechanism is crucial (Li et al., 2021). Such a system should ensure rewards accurately reflect contributions, building trust in the reward-punishment framework (Ryan and Deci, 2020) and mitigating the inhibitive effects of amotivation.

#### Extrinsic regulation-material: a positive yet cautionary influence

The material dimension of extrinsic regulation showed a significant positive association with active voice behavior, confirming that tangible rewards can reduce the perceived “risk cost” of voice, especially among non-tenured nurses (Fedele, 2018). However, relevant research (Fischer et al., 2019) demonstrates that long-term excessive reliance on material rewards and punishments may undermine nurses’ intrinsic motivation, leading to the so-called “crowding-out effect”—where extrinsic incentives weaken the intrinsic drive (e.g., passion for improving patient care) that originally fuels proactive voice. In this study, despite the positive association of the material dimension with voice behavior, the direct positive impact of social rewards and punishments (e.g., public recognition, career advancement) was not significant. This is likely due to contextual factors (e.g., junior nurses’ deference to senior staff in Guangxi’s hierarchical workplace norms, which reduces the motivational impact of public praise) and methodological limitations. Nevertheless, theoretically, social rewards and punishments are crucial for satisfying nurses’ needs for social belonging and career development, and thus should have a potential positive impact on voice behavior (Lee et al., 2022). Additionally, it is noteworthy that external pressure from controlled motivation (e.g., over-reliance on material rewards as a mandatory “incentive” rather than a supplementary one) may inhibit the sustainability and depth of voice behavior, implying that managers need to carefully consider the comprehensive impact of reward and punishment measures on nurses’ long-term work motivation and voice behavior when adopting them.

When formulating reward and punishment measures, nursing managers need to carefully weigh the proportion of material rewards and social rewards and punishments (Feng et al., 2024). Although material rewards may significantly boost nurses’ work enthusiasm in the short term (Fedele, 2018), long-term excessive reliance on such rewards may weaken nurses’ intrinsic motivation, affecting their intrinsic love and responsibility for their work (Deci and Ryan, 2000). Conversely, social rewards and punishments, such as public praise and career advancement, can satisfy nurses’ higher-level needs for social belonging, professional identity, and career development, thereby more effectively stimulating their voice behavior and work engagement (Ayalew et al., 2021; Liang et al., 2012).

Notably, in our nurse sample, the material and social dimensions of extrinsic regulation exhibited significant independence and variability—reflecting diverse motivational needs: some nurses (e.g., non-tenured staff) prioritize material stability, while others (e.g., mid-to-senior title nurses) value social satisfaction more (Abu Yahya et al., 2019). This diversity necessitates a deep understanding of individual needs to design precise incentives—for example, pairing targeted material rewards with low-pressure social recognition (e.g., private feedback) for non-tenured nurses, while prioritizing career advancement-linked social rewards for senior nurses.

#### Other motivation dimensions: conceptual clarity and contextual constraints

The moderate correlation between introjected regulation and extrinsic-social regulation (*r* = 0.670) did not raise multicollinearity concerns (VIF < 10), yet neither dimension demonstrated significant predictive power. This can be explained from two perspectives: first, the externally pressured nature of introjected regulation (e.g., voicing to avoid criticism) is unlikely to sustain the initiative and depth of voice behavior (Deci and Ryan, 2000); second, the effectiveness of extrinsic-social regulation may be constrained by workplace hierarchy norms (e.g., junior nurses’ deference to senior staff could diminish the motivational impact of public recognition) (Zhang et al., 2024). These findings suggest that future management strategies should design low-pressure social reward formats, such as anonymous team appreciation or cross-hierarchical communication meetings.

The non-significant relationship between certain motivational factors and voice behavior profiles may be explained by the organizational context. Specifically, in environments with high power distance, such as healthcare settings in hierarchical cultures, the effect of individual motivation may be attenuated by organizational factors. This aligns with Şahin et al. (2021), who found that power distance negatively influences voice behavior, suggesting that organizational hierarchy can override individual motivational factors.

### Non-significant relationships

Several variables exhibited non-significant relationships with voice behavior profiles, which merit further explanation. First, gender did not predict profile membership, likely due to the extreme gender imbalance in the sample (94.3% female). This homogeneity reduces statistical power to detect potential gender differences, which may be more pronounced in samples with balanced gender representation (Şahin et al., 2021). Second, hospital type showed no significant effect, possibly because both tertiary and secondary hospitals in the study had established basic voice feedback mechanisms (e.g., suggestion boxes, regular staff meetings), minimizing differences in organizational support for voice (Karataş and Çankır, 2023). Third, extrinsic regulation-social did not emerge as a significant predictor, which may be attributed to hierarchical workplace norms in Guangxi’s healthcare settings: junior nurses tend to defer to senior staff, reducing the motivational impact of public recognition or social rewards (Zhang et al., 2024; Şahin et al., 2021). These non-significant results highlight the importance of contextual factors (e.g., sample composition, organizational culture) in shaping the relationship between motivation and voice behavior.

### Evidence-based management strategies rooted in transformational leadership

Drawing on transformational leadership theory (Bass, 1995)—which centers on idealized influence, intellectual stimulation, individualized consideration, and inspirational motivation—we propose tailored, evidence-informed strategies for each nurse voice behavior profile (Rapin et al., 2022).

#### For conservative type (C1): alleviate motivation suppression via “individualized consideration”

Conservative nurses face core barriers of occupational insecurity and hierarchy-induced silence, and strategies prioritize transformational leadership’s “individualized consideration” to address these pain points while leveraging perceived organizational support (Xia et al., 2023). Given that intrinsic motivation is crucial for stimulating work enthusiasm (a key finding from our analysis), nursing managers should provide targeted career development opportunities (e.g., skill training linked to promotion) and modest work autonomy (e.g., letting nurses schedule their daily patient follow-up tasks) to comprehensively stimulate intrinsic motivation—addressing the root of amotivation in this group. To mitigate stress-driven amotivation further, offering confidential one-on-one counseling and monthly stress-relief workshops (Pu et al., 2024) supports nurses’ well-being and helps them convert latent concerns into constructive voice. For hierarchy-related silence, launching an anonymous digital feedback platform (e.g., a WeChat mini-program) themed around “team safety” (e.g., “suggestions to improve patient handoff processes”)—complemented by physical “nurse suggestion boxes” in ward areas—frames voice as a “collective responsibility,” aligning with local collectivist values and mitigating junior nurses’ deference to senior staff (Han and Xu, 2024); this multi-channel approach strengthens their sense of participation while reducing the perceived “risk cost” of speaking up. Simultaneously, establishing a positive feedback system (e.g., weekly team meetings acknowledging even small constructive suggestions) ensures timely and fair recognition of contributions, enhancing job satisfaction and organizational belonging (Feng et al., 2024; Xia et al., 2024).

#### For balanced risk-taker type (C2): resolve motivational contradictions via “intellectual stimulation”

Balanced Risk-Takers require low-risk interventions to convert intrinsic drive into actionable voice. Since intrinsic motivation and identified regulation jointly drive work enthusiasm, managers can increase the interest and challenge of work content (e.g., assigning them to lead small “care process optimization” sub-tasks) to nurture intrinsic motivation, while explaining how these tasks align with nursing professionalism to reinforce identified regulation. Establishing a transparent, merit-based reward mechanism—where rewards tie to voice quality (e.g., “adopted suggestions that enhance care efficiency earn 5% performance bonuses”) rather than quantity—ensures rewards accurately reflect nurses’ actual contributions (Li et al., 2021), building trust in the reward-punishment system (Ryan and Deci, 2020) and reducing amotivation from “unfair treatment.” Complementing this with low-pressure social recognition (e.g., one-on-one acknowledgment from supervisors instead of public praise) avoids triggering discomfort from hierarchical norms while satisfying their need for social validation (Lee et al., 2024); pairing this recognition with a positive feedback system (e.g., written notes of appreciation for thoughtful suggestions) further strengthens organizational belonging, helping resolve their motivational conflict.

#### For transitional type (C3): strengthen identified regulation via “idealized influence”

Transitional nurses act as “change bridges” between conservative and active voice behaviors. Since identified regulation motivates nurses to engage in professional behaviors aligned with self-concept, managers should encourage this group to participate in department management and decision-making (e.g., inviting them to join monthly “ward improvement meetings”) to enhance their autonomy, sense of responsibility, and professional identity; this aligns with our finding that identified regulation strengthens proactive voice. Developing a “Preceptor Voice Workshop”, where nurses teach trainees to both identify care gaps and propose feasible solutions (e.g., “how to suggest workflow tweaks during clinical teaching”), further ties their preceptorship role to proactive voice, reinforcing professional identity and deepening identified regulation (Chen et al., 2012). Department managers can model constructive voice by sharing “voice success cases”(e.g., “a Transitional nurse’s suggestion reduced catheter-related infections by 20%”) in team meetings—framing voice as a marker of “professional maturity” to align with their focus on team stability (Balay-Odao et al., 2022)—and complement this with regular nurse forums to provide structured platforms for opinion exchange, strengthening their sense of participation and belonging (Schaufeli and Bakker, 2004). Additionally, designating “team optimization forums” aligns with collectivist culture, positioning voice as a shared responsibility to reduce resistance to change while sustaining their “steady transition” behavior.

#### For challenging type (C4): sustain autonomous motivation via “inspirational motivation”

Challenging nurses serve as organizational innovators, and strategies prioritize transformational leadership’s “inspirational motivation” to maintain their drive, integrating insights into intrinsic motivation and identified regulation. As intrinsic motivation not only stimulates work enthusiasm but also promotes innovative problem-solving, managers should provide diversified career development opportunities (e.g., fellowships in nursing innovation, advanced clinical training) and greater work autonomy (e.g., letting them design new patient care protocols) to sustain intrinsic motivation—supporting their adoption of innovative methods to address work challenges (Wei et al., 2019). Encouraging deeper participation in department management and decision-making (e.g., appointing them to core nursing quality committees, such as “care innovation steering groups”) aligns with their identified regulation motivation, further enhancing professional identity and reinforcing proactive voice. Granting leadership roles in cross-departmental projects (e.g., piloting smart nursing tools) leverages their experience to spread proactive voice norms, while establishing a “nursing innovation archive”—where impactful suggestions (e.g., those reducing patient readmission rates) are formally documented and linked to career advancement (e.g., promotion prioritization)—validates their role as “change leaders.” Regular nurse forums and digital feedback platforms (in addition to their management roles) provide additional channels for them to express opinions, and a positive feedback system (e.g., organizational awards for “most impactful voice contributions”) ensures timely recognition of their innovations, sustaining autonomous motivation and organizational belonging (Feng et al., 2024; Schaufeli and Bakker, 2004).

## Conclusion

In this research, latent profile analysis was employed to classify nurses’ voice behavior and explore the differences in influencing factors among different categories as well as the relationships with various dimensions of their work motivation. The results revealed that nurses generally exhibited high levels of work motivation, with their voice behavior being above average. Nurses showed moderately high voice behavior and high work motivation; autonomous motivation strongly predicted active voice, while amotivation inhibited it. Extrinsic material rewards had positive but secondary effects, and social rewards were constrained by hierarchy.

Nursing administrators should adopt differentiated strategies: mitigate amotivation for Conservatives, resolve conflicts for Balanced Risk-Takers, strengthen identity for Transitionals, and sustain autonomy for Challengers. Prioritize social rewards, use material incentives cautiously, and establish fair evaluation mechanisms.

This study provides a motivation-based framework for targeted interventions to promote voice, enhance patient safety and care quality. Future longitudinal/cross-cultural research is needed to verify findings.

### Relevance for clinical practice

The findings of this study underscore the critical role of cultivating autonomous motivation among nurses in promoting proactive voice behavior, which is vital to enhancing patient safety and improving the quality of care. Nursing managers should adopt tailored strategies according to nurses’ motivational profiles, including providing opportunities for professional development, strengthening perceived organizational support, and implementing equitable reward systems. Furthermore, establishing structured platforms for voice expression--such as regular forums or digital feedback mechanisms--can enhance communication and foster innovation within healthcare teams, thereby contributing to a more responsive and effective clinical environment.

### Limitations and future research

While this study offers valuable insights, it is not without limitations that should be acknowledged. First, geographical and sample limitations: The sample was restricted to six hospitals in Guangxi Province, China, and the overrepresentation of female nurses (94.3%) and tertiary hospital staff (90.0%) may limit the generalizability of results to other regions or healthcare settings (e.g., community hospitals, male nurses). Additionally, the relatively small sample size of the Conservative profile (*n* = 38, 5.42%) may affect the stability of multinomial logistic regression results, as small subgroups are more vulnerable to sampling error. Second, research design limitations: The cross-sectional design precludes causal inferences between work motivation and voice behavior. Third, unmeasured variables: Key contextual factors such as organizational culture (e.g., power distance), leadership style (e.g., transformational leadership), and implicit voice theories were not fully integrated into the model, despite their known influence on voice behavior. Fourth, data collection limitations: Reliance on self-reported measures may introduce social desirability bias, as nurses may overreport positive voice behavior or underreport amotivation. Fifth, motivation dimension limitations: The study did not explore the interactive effects of different motivation dimensions, which may provide deeper insights into motivational mechanisms.

To address the limitations of this study and advance the field, several directions for future research are proposed. First, methodological improvements: Adopt longitudinal designs to track the dynamic relationships between work motivation, voice behavior profiles, and outcomes (e.g., patient safety, care quality) over time, enabling causal inferences. Use stratified random sampling to include diverse regions (e.g., eastern/western China), hospital types (e.g., community hospitals, specialized hospitals), and demographic groups (e.g., more male nurses, primary-level nurses) to enhance generalizability. Incorporate mixed-methods research (e.g., quantitative surveys + qualitative interviews) to explore the lived experiences of nurses in different voice profiles. Second, variable expansion: Integrate contextual variables such as organizational culture (power distance, psychological safety), leadership style (transformational leadership), and implicit voice theories into the model to explore their moderating/mediating effects. Examine the interactive effects of motivation dimensions and their joint impact on voice behavior. Explore potential mediating variables such as thriving at work and self-efficacy to unpack the mechanisms linking motivation to voice. Third, intervention research: Develop targeted interventions for different voice profiles—for example, implicit voice theory training for Conservatives (C1) to reduce defensive silence, and autonomous motivation enhancement programs for Transitional nurses (C3) to promote active voice. Validate these interventions through randomized controlled trials (RCTs) to assess their effectiveness in improving voice behavior and care quality.

## Data Availability

The datasets utilized and/or analyzed in this study are available upon reasonable request from the corresponding author. Requests to access the datasets should be directed to JH, 202321163@sr.gxmu.edu.cn.

## Footnotes

1
https://www.wjx.cn/
